# The structure of ORC–Cdc6 on an origin DNA reveals the mechanism of ORC activation by the replication initiator Cdc6

**DOI:** 10.1038/s41467-021-24199-1

**Published:** 2021-06-23

**Authors:** Xiang Feng, Yasunori Noguchi, Marta Barbon, Bruce Stillman, Christian Speck, Huilin Li

**Affiliations:** 1 Department of Structural Biology, Van Andel Institute, Grand Rapids, MI USA; 2 DNA Replication Group, Institute of Clinical Sciences, Faculty of Medicine, Imperial College London, London, UK; 3 MRC London Institute of Medical Sciences (LMS), London, UK; 4 Cold Spring Harbor Laboratory, Cold Spring Harbor, NY USA

**Keywords:** DNA, Cryoelectron microscopy

## Abstract

The Origin Recognition Complex (ORC) binds to sites in chromosomes to specify the location of origins of DNA replication. The *S. cerevisiae* ORC binds to specific DNA sequences throughout the cell cycle but becomes active only when it binds to the replication initiator Cdc6. It has been unclear at the molecular level how Cdc6 activates ORC, converting it to an active recruiter of the Mcm2-7 hexamer, the core of the replicative helicase. Here we report the cryo-EM structure at 3.3 Å resolution of the yeast ORC–Cdc6 bound to an 85-bp *ARS1* origin DNA. The structure reveals that Cdc6 contributes to origin DNA recognition via its winged helix domain (WHD) and its initiator-specific motif. Cdc6 binding rearranges a short α-helix in the Orc1 AAA+ domain and the Orc2 WHD, leading to the activation of the Cdc6 ATPase and the formation of the three sites for the recruitment of Mcm2-7, none of which are present in ORC alone. The results illuminate the molecular mechanism of a critical biochemical step in the licensing of eukaryotic replication origins.

## Abstract (web summary)

Eukaryotic DNA replication is mediated by many proteins which are tightly regulated for an efficient firing of replication at each cell cycle. Here the authors report a cryo-EM structure of the yeast ORC–Cdc6 bound to an 85-bp ARS1 origin DNA revealing additional insights into how Cdc6 contributes to origin DNA recognition.

## Introduction

Eukaryotic DNA replication is mediated by over 50 different proteins^1–3^. The process is highly regulated^4^, and the general mechanism of replication is well conserved from yeast to humans^5–7^. DNA replication primarily involves two phases of the cell cycle: establishment or licensing of potential origins of DNA replication occurs during the G1 phase and then in a temporally ordered pattern during S phase, each origin is activated in a controlled manner, resulting in the entire genome being duplicated once^4,6,8^. The initiation of eukaryotic DNA replication is a multi-step, ATP-dependent process that starts with the binding of the six-subunit origin recognition complex (ORC; Orc1-6) around the origin DNA^9–13^. Each of the Orc1-Orc5 subunits contain a winged-helix domain (WHD) plus either an AAA+ domain (ATPases associated with diverse cellular activities) or an AAA-like domain^14–16^. Orc6 differs from the other subunits, and its role varies in different organisms^17,18^. In the budding yeast *Saccharomyces cerevisiae*, there are over 400 origins of DNA replication located on 16 chromosomes and they can function as autonomously replicating sequences (ARSs) when inserted into a plasmid^19^. The functional *S. cerevisiae* origins contain an essential A element that contains an ARS consensus sequence (ACS) and important B1 and B2 elements that contribute to origin recognition and licensing^9,20–23^. *Drosophila* and human replication origins do not have well-defined DNA sequence consensus^24–26^, but a combination of factors including the DNA topology, local chromatin structure, and local histone modifications ultimately determines the location and the timing of replication origins^27–33^.

**Fig. 1:**
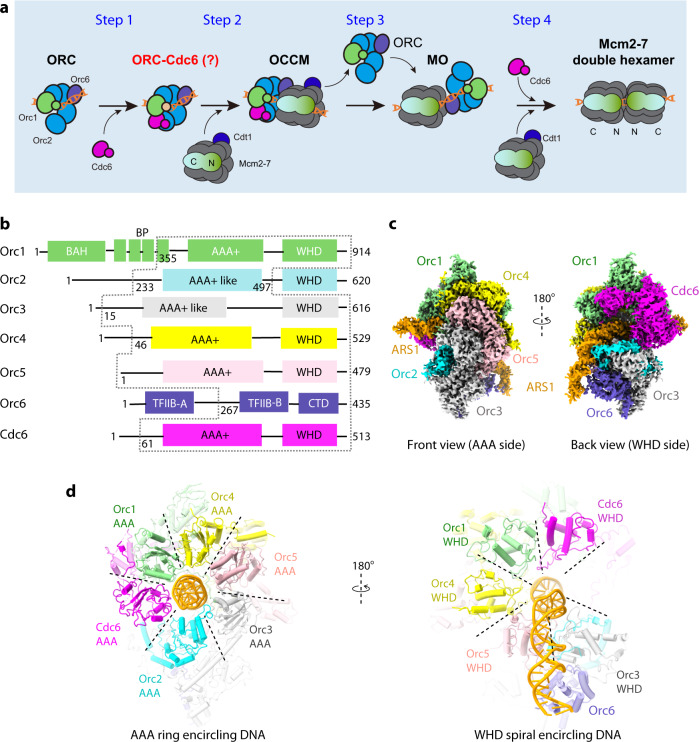
Structure of the *S. cerevisiae* ORC–Cdc6 on the *ARS1* origin DNA. **a** The four-step process of replication origin licensing from ORC–DNA. Only product 1, the assembly of ORC–Cdc6 on origin DNA was not understood at the molecular level. See the “Introduction” for details. **b** Domain organization of Orc1-6 and Cdc6. Regions modeled in the structure are enclosed by dashed gray lines. **c** Cryo-EM 3D map with individual subunits colored. **d** The six AAA+ and AAA-like domains in ORC–Cdc6 nearly symmetrically surround the ACS element of the origin DNA (left), and the five WHD domains spiral around the B1 element (right).

In *S. cerevisiae*, origin licensing occurs by the assembly during G1 phase of a pre-replicative complex (pre-RC) at each potential origin. Pre-RC assembly requires ORC and Cdc6, as well as the Mcm2-7 hetero-hexamer and its chaperone Cdt1, to form a double hexamer (DH) of the Mcm2-7 complex at every origin^2,6,16^. ORC binds to the replication origins throughout the cell cycle, but ORC alone is insufficient for assembly of pre-RCs^10,34–39^. Beginning after anaphase of mitosis, the replication initiator Cdc6 binds to ORC and sets in motion a complex, four-step process that culminates in the establishment of the pre-RC that licenses all potential DNA replication origins^16,40–42^ (Fig. 1a, starting from ORC–DNA). The ORC–Cdc6 complex (product 1) assembles in step 1 around origin DNA and with the help of another replication initiator protein, Cdt1, it recruits the Mcm2-7 hexamer to the origin in step 2. The hexamer is loaded onto the double-stranded DNA (dsDNA), forming product 2, the transient ORC–Cdc6–Cdt1–Mcm2-7 complex (OCCM) that does not require ATP hydrolysis^43,44^. Upon ATP hydrolysis, Cdc6 and Cdt1 are released, leaving the first Mcm2-7 hexamer encircling the origin DNA^45^. During step three, ORC transfers to the opposite N-tier side of the first Mcm2-7 at the B2 element of the origin DNA, forming the Mcm2-7–ORC (MO) complex^44^ (product 3). Finally, in step 4, the MO complex goes on to recruit another Cdc6 to recruit a second Cdt1–Mcm2-7 complex, leading to the eventual assembly of the “head-to-head” (N-face to N-face) Mcm2-7 double hexamer (product 4)^44,46–48^. This completes the origin licensing and the formation of the pre-RC. Pre-RC assembly was reconstituted with purified ORC, Cdc6, Cdt1, and Mcm2-7 on origin DNA and these assembled pre-RCs have been used to initiate DNA replication with purified replication elongation factors and regulatory kinases^49–54^.

Due to considerable structural efforts, the molecular mechanisms for assembly of three of these four products are well understood. For the starting product, ORC–DNA, structural analyses of the yeast, *Drosophila*, and human ORC complexes^14,55–59^, including a high-resolution structure of *S. cerevisiae* ORC bound to the *ARS305* origin DNA^60^, have established ORC architectures. Unlike the yeast ORC, human ORC is assembled and disassembled during the cell division cycle and there is considerable plasticity in the structures of both the *Drosophila* and human ORC^56,58,59^. The yeast ORC recognizes the *ARS305* origin via a stretch of positively charged amino acids within Orc1 and via an α-helix in Orc4 and a loop in Orc2, the latter two protein structures being specific for a small clade of *S. cerevisiae*-related yeast species that contain DNA sequence-specific origins of DNA replication^60–62^. For step 2, in vitro capture and structural analyses of the OCCM intermediate have shown that the Mcm2-7 loading process by ORC–Cdc6 primarily involves one set of the WHDs of ORC–Cdc6 interacting with another set of WHDs at the C-terminal regions of Mcm2-7 subunits, and that DNA enters the Mcm2-7 ring by passing through a side gate between Mcm2 and Mcm5^43,62–65^. For step 3, the capture of the MO intermediate has revealed that the second Mcm2-7 hexamer is loaded by ORC–Cdc6 assembled on the B2 element of origin DNA and bound to the N-tier side of the first Mcm2-7 hexamer^44^. This step requires ATP hydrolysis by the Mcm2-7 hexamer and releases Cdc6 and Cdt1^45,48^. Finally, for step 4, in vitro reconstitution and structural analyses showed that the N-terminal Mcm2-7 peptides interlock to form the partially staggered, highly stable, Mcm2-7 double hexamer and with dsDNA inside the central channel that is slightly bent, but not yet unwound^49,50,66–69^.

A knowledge gap is the detailed, high-resolution structure of the ORC–Cdc6 complex bound to origin DNA that could inform molecular details about steps 1 and 2 in pre-RC assembly (Fig. 1a). Cdc6 is a key initiation factor^41,42,70^ and mutations that impair the Cdc6 ATPase activity are hypomorphic or lethal^34,41,71–74^. However, Cdc6 alone has no ATPase activity, so how the Cdc6 ATPase is activated is not known^75^. Cdc6 activates ORC and induces conformational changes, leading to enhanced origin specificity and an extended ORC region of DNA on both ends of the origin that interacts with protein^14,55,75,76^. For example, Cdc6 binding to ORC on origin DNA greatly extends the length of DNA that is protected from deoxyribonuclease 1 (DNase 1) digestion, a so-called footprint, compared to the length of origin DNA protected by ORC alone^14,75^. However, the nature of these structural changes and how Cdc6 influences ORC–DNA interactions at the origin has remained unclear. The OCCM structures do contain ORC and Cdc6, but this structure includes the assembly of the first Mcm2-7 hexamer during step-2 and thus ORC, Cdc6, and origin DNA may have different configurations than the ORC–Cdc6 complex bound to DNA^62,63^. Here we present a high-resolution structure of ORC–Cdc6 in complex with the *ARS1* origin DNA. The structure explains the binding-induced ATPase activation mechanism of Cdc6, how a single molecule of Cdc6 extends the DNA footprint at both the ends of the origin, and how Cdc6 induced conformational changes converts ORC into an active Mcm2-7 loader. The work fills an important gap in the mechanistic understanding of the replication origin licensing and activation. During peer review of this manuscript, the structure of the *Drosophila* ORC–Cdc6 bound to dsDNA was published^77^. Therefore, we compared the DNA-binding mode of these two structures.

## Results and discussion

### In vitro reconstitution and cryo-EM of the ORC–Cdc6 complex on *ARS1* origin DNA

To reconstitute the ORC–Cdc6 complex, we used an 85 bp fragment of the *ARS1* origin DNA containing the A, B1, and B2 elements. Three components were mixed at a molar ratio of 1 ORC:1.25 DNA:6.4 Cdc6 in the presence of 5 mM ATPγS under reaction conditions similar to the previously established^14,75^ ones. Because Cdc6 binding is known to extend the ORC nuclease protection footprint^14^, we used a long DNA in order to capture all potential protein–DNA interactions with ORC–Cdc6. The *ARS1* DNA was 7 bp longer in front of the A element and 6 bp longer following the B1 element than the *ARS305* DNA used in the structural study of ORC–DNA (Supplementary Fig. 1a–c)^60^. Furthermore, we did not use any cross-linking agent in this study (glutaraldehyde was used to cross-link the ORC–DNA structure)^60^. Cryo-EM grids were screened extensively for optimal sample vitrification conditions. The best conditions led to thin ice and high-contrast particle images, but with the problem of preferred particle orientation. Therefore, two datasets were collected, one without tilting and one with the cryo-EM grid tilted by 20°. Image processing and 3D reconstruction of the combined dataset led to two 3D maps of the same ORC–Cdc6–DNA complex at 3.3 and 3.6 Å resolution (Supplementary Figs. 2 and 3). The two 3D maps were essentially the same except that the 3.6 Å map had stronger density for the Cdc6 WHD. The atomic model was then built and refined based on these two maps and by referencing to the available individual structures of ORC and Cdc6^60,62^.

#### Overall structure of the ORC–Cdc6–DNA complex

Among the seven protein subunits of the complex, six (Orc1-5 and Cdc6) contain an AAA+ or AAA+-like domain and a WHD domain^15,59^. Orc6 is the only subunit without an AAA+/AAA−like domain, but it contains two domains that are similar to transcription factor IIB domains (TFIIB-A and TFIIB-B) and a C-terminal helical domain (Fig. 1b)^17^. In the ORC–DNA structure, the AAA+ domains of Orc1-5 form a ring-shaped structure and the Orc2 WHD domain is located in a gap in this ring between the Orc1 and Orc2 subunits. In our 3D maps of the ORC–Cdc6–DNA complex, the Orc2 WHD was pushed out of the AAA+ ring and was flexible and hence not readily visible. The Orc2 WHD was replaced by Cdc6 that now completed the hexameric ring of Orc1-5 and Cdc6 AAA+ domains (Fig. 1b, c). Also disordered in our structure were the Orc1 BAH domain and the Orc6 TFIIB-A domain. The liberation of the Orc2 WHD makes it available for subsequent recruitment of the Mcm2-7 hexamer (as discussed below).

The six AAA+−containing subunits (Orc1-5 and Cdc6) of ORC–Cdc6–DNA are arranged in a two-tiered, spiral structure that encircles the origin DNA, with the top tier formed by the AAA+ domains and the lower tier by the WHD domains (Fig. 1d, e). On the top tier, the gap between the Orc1 AAA+ and the Orc2 AAA+-like domain vacated by the Orc2 WHD became occupied by the Cdc6 AAA+ domain, and the AAA+ domains of Orc1-5 and Cdc6 form a nearly flat ring that encircles the ACS region of the origin DNA. The six WHDs were domain-swapped with respect to their associated AAA+ domains, as observed in both the *S. cerevisiae* and the *Drosophila* ORC structures^56,60^. On the lower tier, the six WHDs assemble a spiral that wrapped around a longer segment of DNA. Because the interaction of the Cdc6 WHD with DNA was visualized in the 3.6 Å map but not in the 3.3 Å map, the data suggest that the Cdc6 WHD interaction was transient, and that this domain must be partially flexible. The flexibility of the WHDs of Cdc6 and Orc2 is likely to provide them the freedom to engage with the incoming Mcm2-7 hexamer during the OCCM assembly, where these domains are stabilized by the multiple WHDs of the Mcm2-7^62^.

ORC binding to origin DNA is an ATP-dependent event^10^. Indeed, four ATPγS molecules and their associated Mg^2+^ were identified in the AAA+ domains of Orc1, Orc4, Orc5, and Cdc6 (Supplementary Fig. 4). Orc2 and Orc3 have the AAA fold, but their sequences have diverged and they do not have ATPase activity. Accordingly, no ATPγS molecules were observed in these two subunits. The four ATP binding sites in ORC–Cdc6 are similar to those found when ORC–Cdc6 engages Cdt1-bound Mcm2-7 in the OCCM structure^62^, and with the ATP molecules observed in the ATPase active form of human ORC–DNA^57,58^. This observation is consistent with the knowledge that the recruitment of Cdc6 to ORC, as well as the subsequent recruitment of Mcm2-7 to ORC–Cdc6, depends on ATP binding, but not on ATP hydrolysis^45,62,78^.

#### The *ARS1* origin is held by ORC–Cdc6 in a configuration similar to that of the *ARS305* origin by ORC

**Fig. 2:**
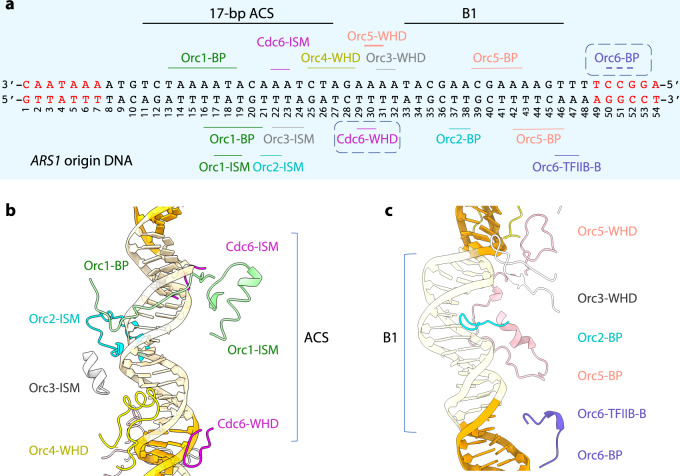
The footprint of ORC–Cdc6 on the *ARS1* origin DNA. **a** The DNA-binding modules in each subunit are labeled around the DNA sequence: the initiator-specific motif (ISM) of the AAA+ domains, the β-hairpin or/and HTH-motif of the WHD domain, and the basic residue patch (BP). Nucleotides in red are the extended footprint in ORC–Cdc6 relative to ORC alone. **b** Structure of the DNA-binding modules around the ACS element. **c** Structures of DNA-binding modules around the B1 element of the origin DNA.

An atomic model was built for 50 out of the total of 85 bp of the *ARS1* DNA in the 3D map. The resolved DNA region included both the A element containing the ACS and the B1 element, as well as 6 nucleotides preceding, and 6 nucleotides following the A and B1 elements, respectively (Fig. 2a). Therefore, the ORC–Cdc6 complex bound and stabilized 50 bp of the *ARS1* origin DNA, which is 9 bp longer than that of ORC alone, which stabilized 41 bp of the *ARS305* origin. Like in the ORC–DNA structure, the origin dsDNA was not straight within ORC–Cdc6. The DNA bending point was around the 35th nucleotide, at the start of the B1 element. Therefore, the B1 element and the sequence containing the B2 element have a bend of about 50° toward Orc6 with respect to the A element DNA. This bend could explain why there exists a prominent DNase 1 hypersensitive site in the footprints when ORC and ORC–Cdc6 bind to origin DNA^10,14^. We note that the B2 element is the loading site for the Mcm2-7 hexamer and thus the bend would place the Mcm2-7 loading site in a specific location with respect to ORC and Cdc6^20,79^.

The bent conformation of the DNA was stabilized by interactions with the basic residues in Orc2, Orc5, and Orc6. The DNA thus seems to be spring-loaded by ORC–Cdc6, such that when these binding elements are released, the DNA outside the central channel will straighten and pass through the entry gate between Mcm2 and Mcm5 in the Mcm2-7 hexamer^63^. The DNA bending point and the bending angle in the ORC–Cdc6–DNA structure are the same as in the ORC–DNA structure^60^. The ORC subunits interact with the origin DNA via three different modules: the initiation-specific motifs (ISMs) in the AAA+ domains, the hairpin or/and inserted helix in the WHDs, and the loops with basic charged residues patches (BP) (Fig. 2b, c)^60^. Most ORC-mediated interactions with DNA are retained in the ORC–Cdc6–DNA structure, except for the flexible Orc2 WHD (Fig. 2a–c and Supplementary Fig. 1b, c). Although the ORC–DNA structure used the *ARS305* origin while our ORC–Cdc6–DNA structure used the *ARS1* origin, the similarity in overall DNA configuration and the specific interactions in the two structures suggest that ORC likely recognizes and binds the hundreds of distinct replication origins with a similar mechanism.

#### How Cdc6 expands the ORC DNase footprint at both ends of the origin DNA

**Fig. 3:**
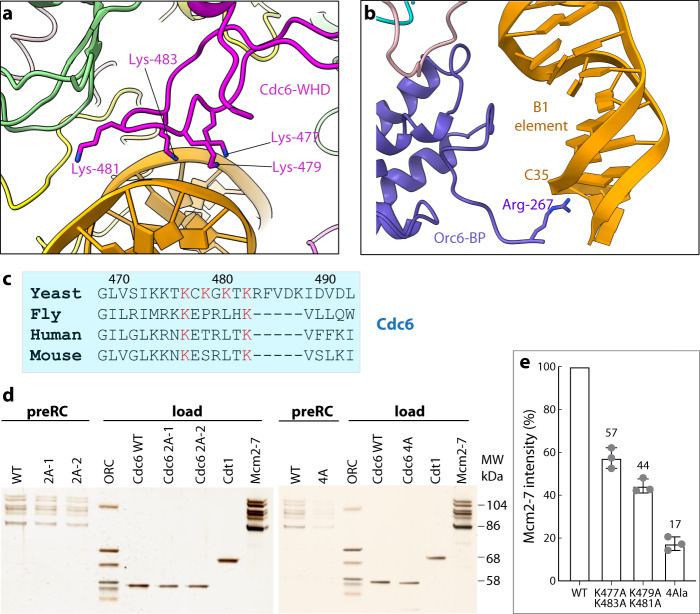
The yeast Cdc6 increased the binding interface to the origin DNA. **a** DNA binding β-hairpin structure in the Cdc6 WHD. The four lysine residues contacting DNA are shown as sticks. **b** The new DNA binding Arg-267 in the Orc6-BP in the ORC–Cdc6 structure. **c** The first and fourth DNA-interacting Cdc6 β-hairpin lysine residues are conserved in eukaryotes. **d** Pre-RC reaction with Cdc6 wild-type (WT), Cdc6 K477A/K483A (2A-1), Cdc6 K479A/K481A (2A-2), or Cdc6 K477A/K479A/K481A/K483A (4A or 4Ala) was assembled, washed with high salt, and analyzed by silver staining. Purified Orc1 (104 kDa), Mcm5 (86 kDa), Cdt1 (68 kDa), and Cdc6 (58 kDa) are used as molecular weight markers. **e** Mcm5 intensities of the Mcm2-7 double hexamer in (**d**) were quantified by ImageJ (version 1.50i) and the relative Mcm5 intensities were plotted using three independent experiments. The center for the error bar is the average of three measurements. The amount of Mcm5 was used as an indicator for the Mcm2-7 loading efficiency of the WT ORC in partner with the mutant Cdc6. Each reaction was done three times independently. Individual data points are shown as gray dots. Error bars represent standard deviations.

The high-resolution structure of the ORC–Cdc6–DNA reveals new DNA-binding sites in Cdc6 and Orc6 that are absent in the ORC–DNA structure (Fig. 3a, b) and provides an opportunity to examine how Cdc6 contributes to the origin DNA specification. Cdc6 was found to interact extensively with dsDNA in a bipartite manner, with the ISM of its AAA+ domain binding to the middle of the ACS sequence and the WHD hairpin motif binding to the intervening region between the A and B1 elements (Fig. 2a–c and Supplementary Fig. 5a). The positively charged residues K477, K479, K481, and K483 in the Cdc6 WHD hairpin motif interact with the ACS phosphate backbone, but do not insert into the DNA groove, and therefore most likely increases the affinity of the ORC–Cdc6 complex for DNA (Fig. 3a). The interaction is likely a defining feature of Cdc6, because two of the four lysine residues (K477 and K483) are universally conserved among eukaryotes and the non-yeast CDC6 have a conserved arginine at the equivalent position of yeast G480 (Fig. 3c and Supplementary Fig. 6**)**. We found that the two double-alanine Cdc6 mutants (K477A/K479A and K481A/K483A) had reduced efficiency in assembling the pre-RC in vitro; they were, respectively, 57% and 44% as efficient as the wild-type Cdc6 (Fig. 3d, e). Combining these double mutants to produce the four-alanine Cdc6 mutant (K477A/K479/K481A/K483A) rendered Cdc6 largely nonfunctional for Mcm2-7 loading, with 17% of the WT efficiency. This is likely due to redundancy in the lysine residues that interact with the origin DNA and two can suffice but altering all four abrogates pre-RC assembly. The in vitro assay highlights the functional importance of the positively charged residues in the Cdc6 WHD hairpin.

Two Cdc6 molecules are normally required for efficient assembly of Mmc2-7 double hexamers during pre-RC assembly^44,45^. Cdc6 ATPase activity is required for removal of the Cdc6 protein from the loaded Mcm2-7 during the pre-RC assembly reaction and a Cdc6 ATPase mutant (E224Q) loads much less Mcm2-7 protein in vitro, although it can form some double hexamers^80^. We suggest that the Cdc6 ATPase activity is required for removal of the first and second Cdc6 proteins during pre-RC assembly. In the absence of Cdc6 ATPase activity, loading of the second Mcm2-7 is less efficient because the first Cdc6 remains bound to ORC, necessitating a completely new ORC–Cdc6 complex to load the second Mcm2-7 hexamer. Such a scenario would explain why Cdc6 ATPase mutants cannot progress through S phase efficiently, due to inefficient Mcm2-7 double hexamer activity^80^. Because the double and quadruple Cdc6 mutations are distant to the ATPase site, it is unclear if the Cdc6 ATPase is affected, although this is a possibility. Nevertheless, the mutant Cdc6 must form a less stable ORC–Cdc6–DNA complex, which may lead to altered Cdc6 ATPase activity, in a manner perhaps similar to WT ORC–Cdc6 on non-origin DNA, which leads to higher Cdc6 ATPase and a destabilized ORC–Cdc6–DNA complex^75^. Finally, because the Cdc6 WHD hairpin binds to the major bending point between the A and B1 elements of the *ARS1* origin, the Cdc6 mutations may lead to a less-bent DNA configuration, thereby hindering DNA insertion into the Mcm2-7 hexamer^63^.

Cdc6 was shown to extend the DNA footprint of ORC on both ends of the DNA, in front of the A element and following the B1 element^14^. However, Cdc6 in our structure does not make direct contact with the extended DNA in front of the A element. We speculate that the stabilization of this region is an allosteric effect of Cdc6 stabilizing the overall ACS region, perhaps making the double helix stiffer so that a structured helix extends beyond the stable protein interactions sites. Another possibility is that a flexible protein sequence within the intrinsically disordered region (IDR) of Orc1, just amino-terminal to the lysine-rich DNA binding region, binds to this DNA but is not visible in the 3D map. Consistent with this suggestion, in the ORC–DNA structure, a lysine-rich sequence in the intrinsically disordered domain of Orc1 binds to a minor grove in the origin DNA^60^, but this interaction is not visible in the ORC–Cdc6–DNA structure shown here. The IDR of human CDC6 binds to the IDR of human ORC1 and there is also an ORC1-ORC1 self-interaction mediated within its own IDR^81^. If such IDR interactions occur in *S. cerevisiae* by the Cdc6 IDR binding to the Orc1 IDR, concomitant with the movement of the Orc2 WHD, when this may stabilize the DNA in a flexible location that cannot be seen by cryo-EM. At the other end of the origin DNA, the eight nucleotides following the B1 element were stabilized by the non-conserved Orc6 BP (R267), not by Cdc6 **(**Figs. 2a and 3b**)**. This eight-nucleotide sequence was also present in the previous structural study of ORC with the *ARS305* DNA substrate^60^, but this region and the R267 of Orc6 were not stabilized and not resolved in that structure. Our structure shows that Cdc6 binding stabilizes the Orc6 TFIIB-B domain, which in turn binds and stabilizes the eight nucleotides following the B1 element (Supplementary Fig. 5b). These structural observations fit with the previous biochemical study showing that Cdc6 binding expands the DNase 1 footprint of ORC both in front of the A element and following the B1 element^14^ and footprint analysis of yeast origins in vivo, which showed in G2 phase of the cell cycle a compact ORC footprint and an expanded footprint in G1 phase of the cell cycle^82^. Our structure provides a reasonable explanation for how a single protein (Cdc6) could expand the DNA footprint at both ends of the origin DNA, which are separated by a vast distance over 40 bp of DNA. The actual Cdc6 binding is well within the A and B1 element that ORC covers, but the extended stabilized regions of the origin DNA are a result of allosteric effects caused by Cdc6 binding.

#### Comparison with the *Drosophila* ORC–Cdc6–DNA structure

**Fig. 4:**
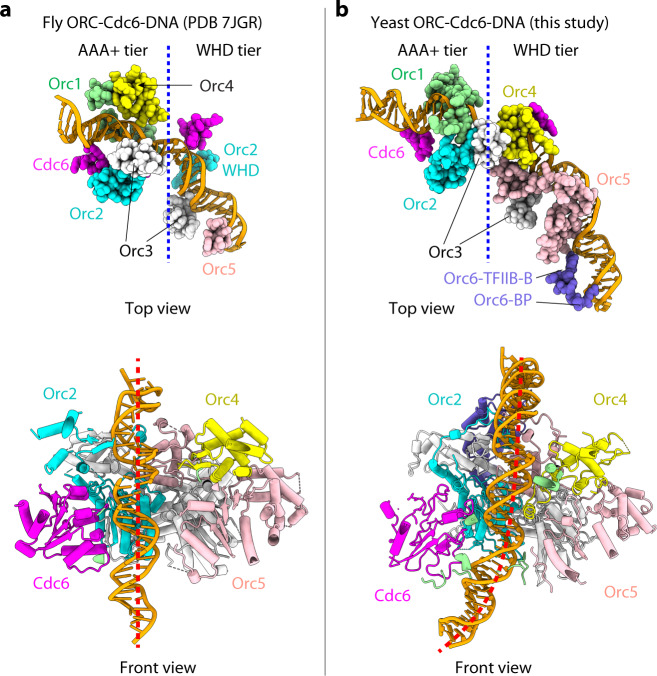
Comparison of the drosophila and yeast ORC–Cdc6 complexes bound to origin DNA of a similar length. **a**, **b** Structure of the fly ORC–Cdc6 bound to an 82 bp AT-rich dsDNA (**a**) and the yeast ORC–Cdc6 bound to the 85 bp ARS1 origin dsDNA (**b**). The upper panels show top view of protein residues (spheres) that are within 4 Å of DNA. The yeast ORC–Cdc6 complex contains extra contacts with DNA by the Orc4 AAA+ domain and the Orc2 WHD domain (unresolved). The lower panels show the DNA binding domains (cartoons) in the complexes in a front view. The fly DNA is straight, but the yeast origin DNA is bent due to the extra DNA-binding motifs present in the yeast structure (Orc1-BP, Orc4-WHD, Orc5-BP, Orc6-TFIIB-B, and Orc6-BP) but absent in the fly structure.

A *Drosophila* ORC–Cdc6 structure solved most recently was bound to an 84 bp dsDNA, which is of similar length to the 85 bp ARS1 DNA used in this study^77^. A side-by-side comparison showed that the *Drosophila* and yeast ORC–Cdc6 complexes are arranged similarly around the dsDNA, indicating a general conservation of the earliest stages of the replication-initiation mechanism. The two structures are best aligned around the bound dsDNA. However, the protein–DNA interface in the yeast structure is more extensive, leading to the longer stabilized DNA in the yeast structure (Fig. 4a, b). Furthermore, the DNA in the *Drosophila* structure passes straight through the ORC–Cdc6 complex while the DNA in yeast structure is tilted as well as bent. Although there exist many DNA-binding motifs that are shared between the two ORC–Cdc6 structures, important differences exist: (1) the yeast Orc2 AAA+ domain is shielded by the Orc1 BP motif and the yeast Orc2-WHD is disordered. (2) The yeast Orc1 BP, Orc4 WHD, Orc5 BP, Orc6 TFIIB-B, and Orc6 BP all interact with DNA, whereas the corresponding *Drosophila* motifs do not (Figs. 2a and  4). (3) The *Drosophila* protein residues mostly interact with DNA backbone, whereas the yeast protein residues frequently insert into the major or minor DNA grooves. These DNA binding differences are consistent with the non-sequence-specific DNA binding of the *Drosophila* structure.

#### Cdc6 drives Orc2 conformational change and the formation of three composite sites for Mcm2-7 loading

**Fig. 5:**
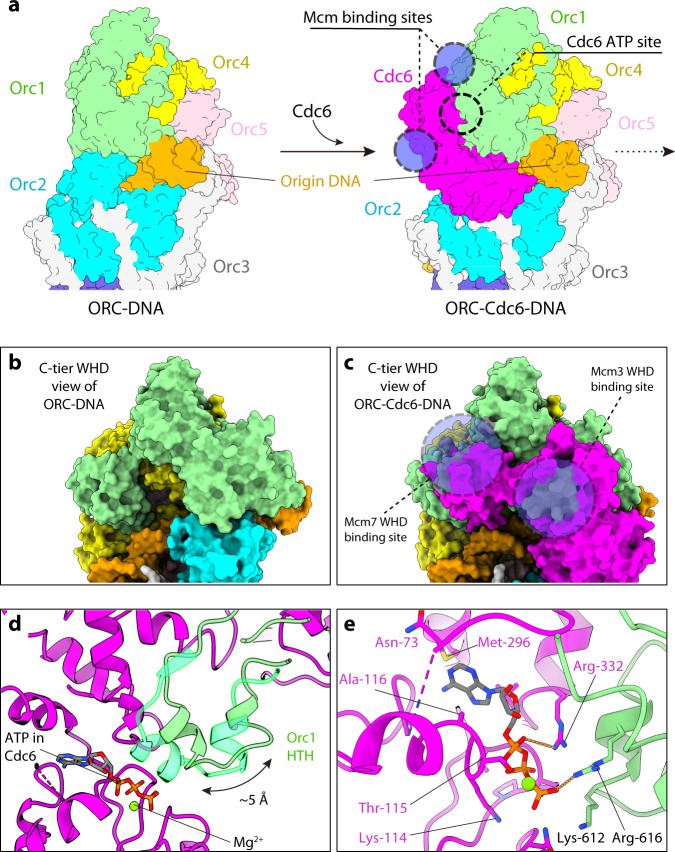
Structural change in Orc1 that accommodates Cdc6 binding and activates the Cdc6 ATPase. **a** Comparison of ORC (left) and ORC–Cdc6 (right) in surface view showing that the binding of the Cdc6 to ORC leads to the assembly of three composite Mcm2-7 binding sites, which are absent in ORC itself. **b**, **c** Enlarged view of panel a showing the absence (**b**) and the presence (**c**) of the binding sites (dashed black circles) for the WHDs of Mcm3 and Mcm7. **d** A short α-helix of Orc1 AAA+ domain shifts by 5 Å upon Cdc6 binding to form the ATP-binding pocket of Cdc6. **e** The Cdc6 ATP binding site is formed by side chains from both Cdc6 and Orc1. Orc1 Arg-616 serves as the arginine finger for the ATP in Cdc6. ATPγS and surrounding residues are shown as sticks.

By comparing the structures of the ORC–Cdc6–DNA and the ORC–DNA complexes^60^, we found that Orc2 had undergone a major conformational change. Specifically, the Orc2 WHD was evicted from the center of the 5-membered AAA+ ring in the structure of ORC–DNA and was replaced by the Cdc6 AAA+ domain, forming the closed ring of six AAA+ domains around the ACS element in the ORC–Cdc6–DNA structure (Figs. 1d and 5a). We compared the new ORC–Cdc6–DNA structure with the same complex within the lower resolution structures of the OCCM complexes. In those complexes, ORC–Cdc6 was transformed to gradually recruit and interact with Mcm2-7 in the semi-attached state (4.3 Å), in the pre-insertion state (8.1 Å)^63^, or in the DNA-inserted state (3.9 Å)^62^. The comparison showed that the Cdc6-binding-associated changes had the net effect of assembling three required Mcm2-7 binding sites in ORC–Cdc6, in particular, the first two sites in ORC–Cdc6 onto which the WHDs of Mcm3 and Mcm7 initially anchor (Fig. 5b, c). None of the three composite Mcm2-7 binding sites in the ORC–Cdc6 complex were present in the ORC–DNA structure (Fig. 5a). Therefore, the ORC–Cdc6–DNA structure provides additional insight into the requirement of Cdc6 for the ORC-dependent recruitment of Mcm2-7^43,62,63^.

#### Orc1 activates the Cdc6 ATPase

Cdc6 ATPase activity is not needed for ORC loading of the Mcm2-7 hexamer, but it is required for ORC–Cdc6 dissociation from the loaded Mcm2-7 hexamer on DNA during pre-RC assembly, for assembly of multiple Mcm2-7 double hexamers and for quality control to remove incorrectly loaded Mcm2-7 hexamers^64,78,80,83,84^. However, Cdc6 alone is inactive and has no ATP hydrolysis activity; the Cdc6 ATPase is activated only when Cdc6 binds ORC^75^. We found that Cdc6 binding shifted a helix-turn-helix (HTH) motif in Orc1 by about 5 Å to form a part of the ATP binding site of Cdc6 (Fig. 5d, e). This Orc1 region interacts with the WHD of Orc2 in the ORC–DNA structure, particularly residues Arg-607 and Gln-608, but upon Cdc6 binding, the HTH moved so that Orc1 residues Arg-616 and Lys-612 join the Cdc6 Walker-A motif to assemble the composite ATPase site of Cdc6 (Fig. 5e). In fact, the Orc1 Arg-616 points to the γ-phosphate and serves as the arginine finger in trans for the ATP molecule in the Cdc6 pocket. The composite ATP site of Cdc6 resembles the Orc1 ATP site formed between Orc1 and Orc4^84^ (Supplementary Fig. 8). However, the ATPγS coordination in Orc4 (between Orc4 and Orc5) and Orc5 (between Orc5 and Orc3) is different, probably a reflection of their unique roles in origin licensing^57,60^. Because the Cdc6 ATPase is required for origin licensing, it is not surprising that the ATP-binding-pocket mutations, such as that of the highly conserved Lys-114 of the essential Walker A motif, leads to growth defects^72^. We suggest that the ORC–Cdc6-assembly-dependent activation of the Cdc6 ATPase prevents futile ATP hydrolysis by free Cdc6 and that the Cdc6 ATPase is turned on only when its activity is needed during pre-RC assembly. The process is likely conserved since the human ORC2 WHD interacts with ORC1 in the ORC2-5 structure and occupies a number of dynamic positions^58^.

#### The Cdc6 WHD undergoes a series of movements during Mcm2-7 loading

**Fig. 6:**
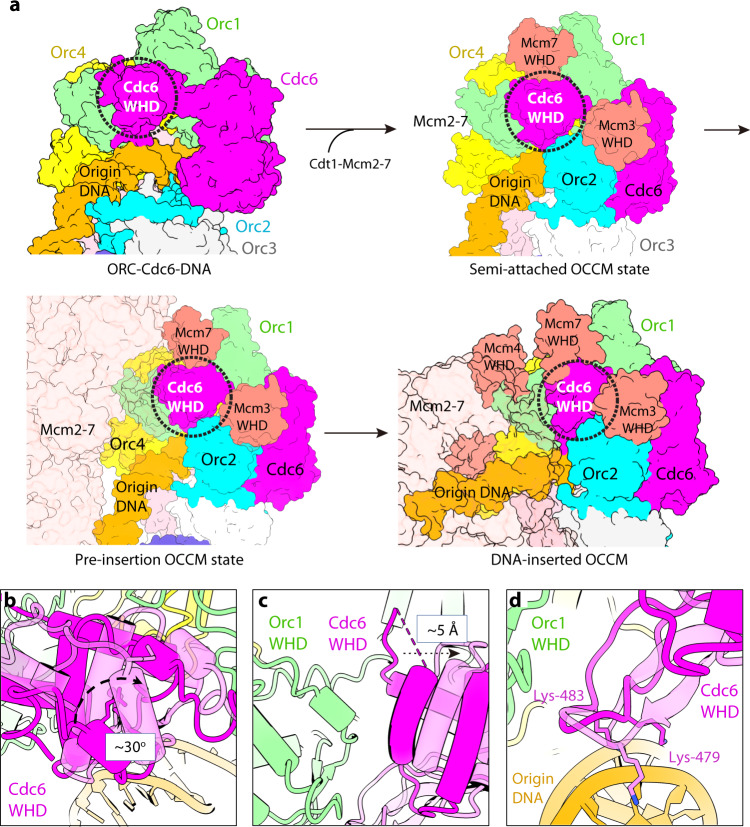
The Cdc6 WHD undergoes multiple conformational changes from ORC–Cdc6 to OCCM during the loading of the Mcm2-7 hexamer. **a** Conformational changes of the Cdc6 WHD during the loading of Mcm2-7 from ORC–Cdc6 to semi-attached OCCM to pre-insertion OCCM and to the DNA-inserted OCCM. The dashed black circle in the last panel encircles the Cdc6 WHD binding region; this region is enlarged in panels (**b**–**d**) and shown in different views. **b**, **c** The Cdc6 WHD rotates (**b**) and shifts (**c**) relative to the Orc1 AAA+ domain. **d** The repositioned Cdc6 WHD in OCCM inserts Lys-479 and Lys-483 into the now straightened ACS DNA groove. In panels (**b**–**d**), the Cdc6 WHD is shown as solid purple in the ORC–Cdc6 and as transparent purple in the OCCM structure.

By comparing the current structure of ORC–Cdc6–DNA with the ORC–Cdc6 structure present in several pre-RC loading intermediates, including the semi-attached and pre-insertion OCCM states and the DNA-inserted OCCM complex (Fig. 6a), we found that the Cdc6 WHD does not move much from the ORC–Cdc6 alone to the semi-attached OCCM. However, Cdc6 WHD is sandwiched between the two first-engaging WHDs of Mcm3 and Mcm7, indicating that the presence of Cdc6 is a prerequisite to Mcm2-7 loading. The Cdc6 WHD largely sustains its position during transition from the semi-attached to pre-insertion OCCM. However, upon transitioning to the DNA-inserted OCCM, the Cdc6 WHD rotates 30° and shifts 5 Å away from the Orc1 WHD (Fig. 6b, c), such that the two WHDs lose their interaction. WHDs of Orc1 and Cdc6 are then stabilized, respectively, by the WHDs of Mcm4 and Mcm7, the double-anchor projected from the Cdt1-bound Mcm2-7 hexamer^63^. In the structure of the DNA-inserted OCCM, the Mcm3 WHD binds at the interface between the Orc2 WHD and the Cdc6 AAA+ domain, while the Mcm7 WHD binds at the interface between the Cdc6 WHD domain and the Orc1 AAA+ domain (Fig. 6a). The movement of Cdc6 WHD has another profound effect on DNA binding. The hairpin loop of the Cdc6 WHD (Lys-477 to Lys-483) moves closer to interact with the phosphate backbone between ACS and B1 element of the ARS1 origin DNA, increasing the hairpin-loop–DNA binding surface from 141 to 165 Å^2^ (Fig. 6d). This interacting DNA region is the pivot around which the B1 element will unbend and straighten to pass through the Mcm2 and Mcm5 gate during the Mcm2-7 loading process^63^. Therefore, during Mcm2-7 loading, the Cdc6 WHD grips on DNA immediately upstream of the B1 element, possibly providing a pivot for B1 and B2 unbending and their precise insertion into the Mcm2-7 central chamber. In the semi-attached and pre-insertion states that precede the fully assembled and DNA-inserted OCCM state, the Cdc6 WHD is positioned as in the ORC–Cdc6–DNA structure^63^. Therefore, the movement of the Cdc6 WHD coincides with DNA insertion into the Mcm2-7 chamber, which occurs after the Mcm2-7 hexamer has been recruited and attached to ORC–Cdc6.

In summary, the ORC–Cdc6 complex bound to the *ARS1* origin DNA has illuminated additional details of how Cdc6 is involved in recruiting the Mcm2-7 hexamer and how its regulated ATPase activity is configured just at the right time during DNA replication. The structure provides a plethora of insights into the origin licensing process, including how Cdc6 transforms the ORC from the passive origin binder into an active platform for recruiting and loading the Mcm2-7 hexamers; how Cdc6 extends the ORC footprint on both sides of the origin; how Cdc6 ATPase is activated by Orc1 via a binding-induced conformational change; and how the Cdc6 WHD alters the DNA-binding mode to facilitate DNA insertion during the Mcm2-7 loading. This structure fills a major gap in our understanding of replication origin licensing.

## Methods

### ORC and Cdc6 expression and purification

ORC was purified as described previously, with minor modifications^85^. Throughout the purification, 1 mM ATP and 5 mM MgCl_2_ were present, but then omitted during the gel filtration with the Superdex 200 16/6 column. The Cdc6 mutant constructs (K477A/K479A, K481A/K483A, and K477A/K479A/K481A/K483A) were ordered from GenScript. Cdc6 was purified as described previously, with minor modifications^14^. The lysis buffer contained 500 mM potassium phosphate.

### In vitro ORC–Cdc6–DNA complex reconstitution

Forward and reverse of 85 bp ARS1 DNA (forward: 5ʹ- GTTATTTTACAGATTTTATGTTTAGATCTTTTATGCTTGCTTTTCAAAAGGCCTGCAGGCAAGTGCACAAACAATACTTAAATAA-3ʹ, reverse: 5ʹ- TTATTTAAGTATTGTTTGTGCACTTGCCTGCAGGCCTTTTGAAAAGCAAGCATAAAAGATCTAAACATAAAATCTGTAAAATAAC-3ʹ) was commercially synthesized by Integrated DNA Technologies (IDT) through the custom DNA oligonucleotides service. The dsDNA was prepared by annealing the two ssDNAs. The annealing buffer condition was 10 mM HEPES-KOH, pH7.5, 1 mM EDTA, and 50 mM NaCl. The system was heated to 95 °C before it was gradually cooled to the room temperature, then kept at –20 °C before use for the complex reconstitution.

For ORC–Cdc6–DNA complex reconstitution, the origin dsDNA (100 μM) was added to the ORC protein solution. The ORC at the final concentration of 1.6 mg/ml (about 4 μM) was mixed with the dsDNA (5 μM). The reaction buffer contained 5 mM DTT, 5 mM ATPγS, and 9% glycerol. The mixture was first incubated for 10 min at the room temperature, then moved onto ice and incubated for 60 min. Next, Cdc6 was added to the reaction mixture, leading to the final concentration of about 1 μM ORC:1.25 μM DNA:6.4 μM Cdc6. The use of excess Cdc6 was to increase the chance of the in vitro assembly of the ternary ORC–DNA–Cdc6 complex. The reaction solution was mixed and incubated for another 60 min on ice. The glycerol in the reconstitution buffer was removed by spin-column centrifugation (Amicon Ultra-0.5 ml centrifugal filter unit). The sample was concentrated to a final volume of about 30 μl and divided into ten 3 μl aliquots and used immediately.

### Pre-RC assemble assay

The pre-RC assemble assay was performed as described^50^ with some modifications. 40 nM ORC, 20 nM Cdc6, 40 nM Cdt1, 40 nM Mcm2-7, and 6 nM 3 kb ARS1 fragment-beads in 50 μl buffer A (50 mM HEPES-KOH (pH 7.5), 100 mM potassium glutamate, 10 mM magnesium acetate, 100 μM zinc acetate, 3 mM ATP, 5 mM DTT, 0.1% Triton X-100, 50 μM EDTA, and 10% glycerol) were incubated for 20 min at 24 °C to form pre-RC complex. Then, after three washes with buffer A with 300 mM sodium chloride to keep only the salt-resistant Mcm2-7 bound to the DNA, the complex was eluted with 1 U of DNase I in buffer A with 5 mM CaCl_2_. The recruited Mcm2-7 were then separated by SDS-PAGE and visualized by silver staining. Each mutant was repeated three times.

### Electron microscopy and data acquisition

After assembly, the ORC–DNA–Cdc6 solution was aliquoted to prepare EM grids for microscopy. For negative staining, a 3 μl aliquot was diluted 10-fold and was applied onto carbon-film-supported copper EM grids (Electron Microscopy Science), washed with buffer, and then stained with 2% uranyl acetate aqueous solution. The negative staining grids were examined in a Tecnai electron microscope (TFS) operated at 120 kV, which was equipped with a 2k × 2k Orius 830 CCD camera (Gatan). For single-particle cryo-EM analysis of ORC–Cdc6–DNA, the undiluted sample was applied three times to glow-discharged holey carbon grids (Quantifoil R1.2/1.3 Copper, 300 mesh). The EM grids were blotted for 3 s after each sample application using a piece of filter paper. Then the grids were plunged into liquid ethane cooled by liquid nitrogen using a FEI Vitrobot Mark IV. A pilot dataset of 945 micrographs was collected in a 200 kV FEI Arctica electron microscope equipped with a K2 summit camera (Gatan) for screening purposes. After 2D and 3D classification, we obtained 21,236 particles. The 3D reconstruction and refinement led to a preliminary 6.5 Å 3D map that confirmed the presence of both origin DNA and Cdc6 in the structure.

We collected two datasets (A and B) during two separate sessions using a TFS Titan Krios electron microscope operated at 300 kV and equipped with a K3 summit camera (Gatan). Dataset A was not tilted. Dataset B was recorded with the sample stage tilted by 20° in order to alleviate a slight preferred orientation problem. Both datasets were acquired with the objective lens defocus range of –1.0 to –2.0 μm at a nominal magnification of 130,000× using SerialEM-3.8.8^86^, with an effective calibrated image pixel size of 0.414 Å. All EM images were recorded in the super-resolution counting and movie mode with a dose rate of 1.5 (dataset A) and 1.9 (dataset B) electrons per Å^2^ per second per frame. A total of 40 frames were recorded in each movie micrograph.

### Image processing

The untilted dataset A contained 18,546 movie stacks. These movies were drift-corrected with electron-dose weighting and 2-fold binned using MotionCor2 (version 1.1.0)^87^. The full dataset was split into four subsets and imported to Relion-3.0^88^. For each subset, the particles were auto-picked and extracted with 4-fold bin. After 2D classification, the “good” 2D class averages with defined structural features were selected and imported into Cryosparc2 (version 2.15.0)^89^ for ab initio 3D model reconstruction. The particle images from the best 3D map with good structural details that were reconstructed from each of the four subsets were merged and converted to the RELION format using UCSF PyEM (version 0.5) (https://github.com/asarnow/pyem). This led to a total of 419,781 particles. Another round of 3D classification was run on the particles before 3D refinement to 3.8 Å density map. Unfortunately, this density map had a strong orientation preference issue.

The tilted dataset B contained 4746 movie stacks that were drift-corrected with MotionCor2 (version 1.1.0) as we did with dataset A. However, the particle selection was done by the Gautomatch (version 0.56) program using projection of the 3D classes obtained from dataset A as the templates. Then the per-particle-based CTF parameters were calculated using GoCTF (version1.1.0), a program specifically developed for processing titled cryo-EM datasets^90^. The subsequent processing steps were the same as with dataset A. We merged the best particles from processing of each subsets in Cryosparc2 (version 2.15.0), and the final dataset had 177,397 particles. Further 3D classification and refinement led to the selection of a subset of 72,000 particles that produced a 3D map with solid density for the Cdc6 WHD at 4.0 Å resolution. CTFrefine and polishing improved this map to the final resolution of 3.6 Å, based on the 0.143 threshold of the gold-standard Fourier shell correlation between the independently constructed 3D “half” maps, with each map using half of the dataset.

The merging of the particles from datasets B and A help solve the orientation preference issue. The final resolution of the 3D map reconstructed from the merged dataset reached 3.3 Å. However, the density of the Cdc6 WHD was weak relative to the 3.6 Å 3D map.

### Model building and refinement

The initial atomic model was built by fitting the published structures of the yeast ORC and Cdc6. Both 3D maps were used in the structural model building because the two maps were superimposable, except that 3.3 Å 3D map had better detail in the ORC–DNA region, while the 3.6 Å 3D map had better density for Cdc6. The template for the ORC–DNA regions was the yeast ORC–DNA complex bound to the *ARS305* origin DNA (PDB ID 5ZR1). The template for the Cdc6 structure was extracted from the structure of yeast OCCM complex (PDB ID 5V8F). Following initial docking, the atomic model was manually rebuilt with the program COOT (version 0.8.9.1)^91^ followed by real-space refinement in the PHENIX program (version 1.14-3260)^92^. Finally, the atomic model was validated using MolProbity (version 4.5)^93,94^. The figures were generated with the UCSF ChimeraX (version 1.0)^95^.

### Reporting summary

Further information on research design is available in the Nature Research Reporting Summary linked to this article.

## Supplementary information

Supplementary information.

Reporting summary.

## Data Availability

The 3D cryo-EM maps of ORC–Cdc6–DNA at 3.3 Å and 3.6 Å have been deposited in the Electron Microscopy Data Bank under accession code EMD-23818 and EMD-23755, respectively. The atomic model based on the two 3D maps has been deposited in the Protein Data Bank under accession code PDB ID 7MCA[10.2210/pdb7MCA/pdb]. All data are available from the authors upon reasonable request.
