# Supplementary material for: The structure of ORC–Cdc6 on an origin DNA reveals the mechanism of ORC activation by the replication initiator Cdc6
Source: Nat Commun. 2021 Jun 23;12:3883. doi: 10.1038/s41467-021-24199-1 (PMC8222357; doi:10.1038/s41467-021-24199-1)
Supplement: Supplementary file 1 — Supplementary information. [file 41467_2021_24199_MOESM1_ESM.pdf]

Supplementary Information

The structure of ORC–Cdc6 on an Origin DNA Reveals the Mechanism of ORC Activation by the Replication Initiator Cdc6

By Feng et al.

This document contains:

Supplemental Table 1
Supplemental Figures 1 – 7

Supplementary Table 1. Cryo-EM data collection, refinement, and validation statistics for the yeast ORC–Cdc6–DNA complex

	ORC–Cdc6–DNA (EMD-23818)	ORC–Cdc6–DNA (EMDB-23755, PDB 7MCA)
**Data collection and processing**		
Microscope	FEI Titan Krios	FEI Titan Krios
Voltage (kV)	300	300
Electron exposure (e^{-} Å^{-2})	60	76
Defocus range (μm)	-1.0 – -2.0	-1.0 – -2.0
Pixel size (Å)	0.828	0.828
Symmetry imposed	C1	C1
Initial particle images (no.)	419,781	177,397
Final particle images (no.)	264,117(combined)	72,846
Map resolution (Å)	3.3	3.6
FSC threshold	0.143	0.143
Map resolution range (Å)	43–3.3	43–3.6
**Refinement**		
Map sharpening B factor (Å)	-118.9	-118.5
Model composition		
Non-hydrogen atoms		24,693
Protein residues		2,760
Nucleotide		100
**R.m.s. deviations**		
Bond lengths (Å)		0.007
Bond angles (°)		0.961
**Validation**		
MolProbity score		1.83
Clashscore		6.04
Poor rotamers (%)		0.47
**Ramachandran plot**		
Favored (%)		91.69
Allowed (%)		8.31
Outliers (%)		0

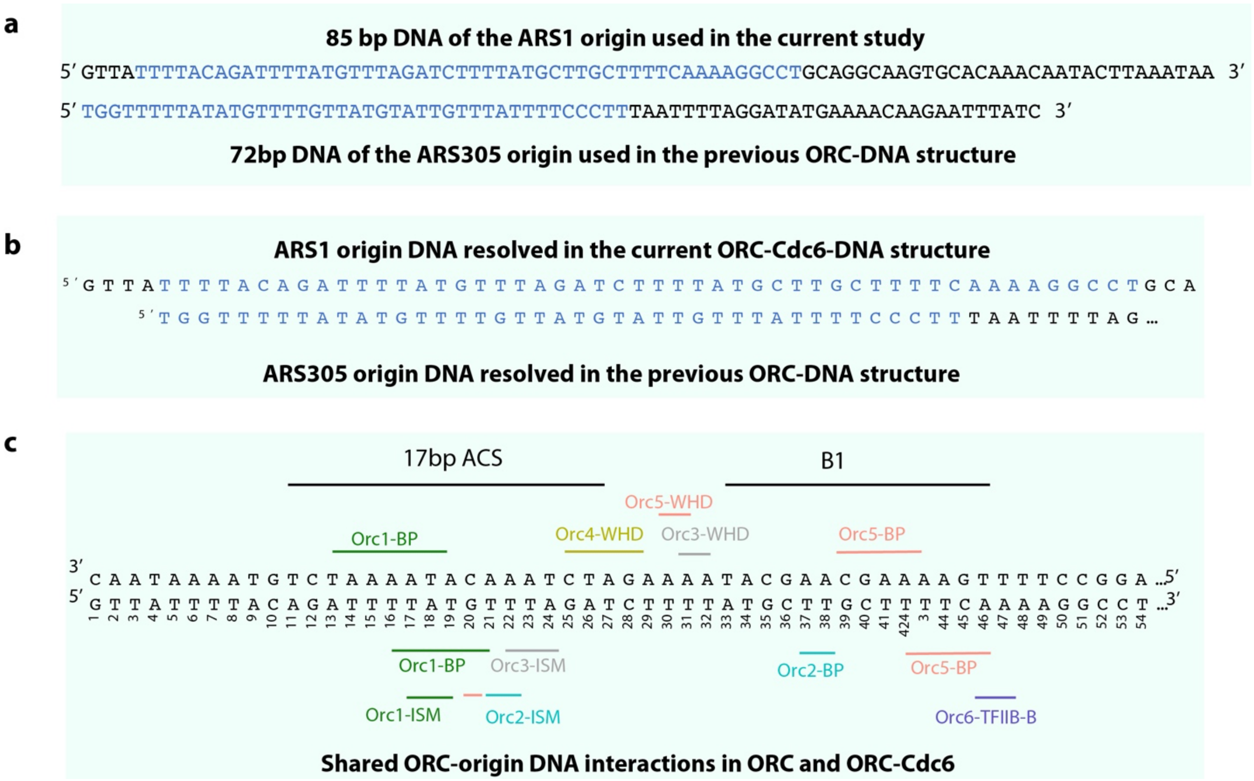

Supplementary Figure 1. ***ARS1* origin DNA used in the current study compared with the *ARS305* origin DNA used in the previous ORC–DNA structural study. a)** The 85-bp DNA sequence used in current ORC–Cdc6–DNA study as compared to the 72-bp ARS305 DNA used in ORC–DNA structure [60]. The observed sequence in the structures are shown in blue. **b**) Alignment of the two origin DNAs by superimposing their respective ORC structures. **c**) The shared protein–DNA interaction regions between ORC and the ORC–Cdc6 structure are marked. This comparison reveals a set of consensus ORC binding sites in the two distinct origins (*ARS1 and ARS305*).

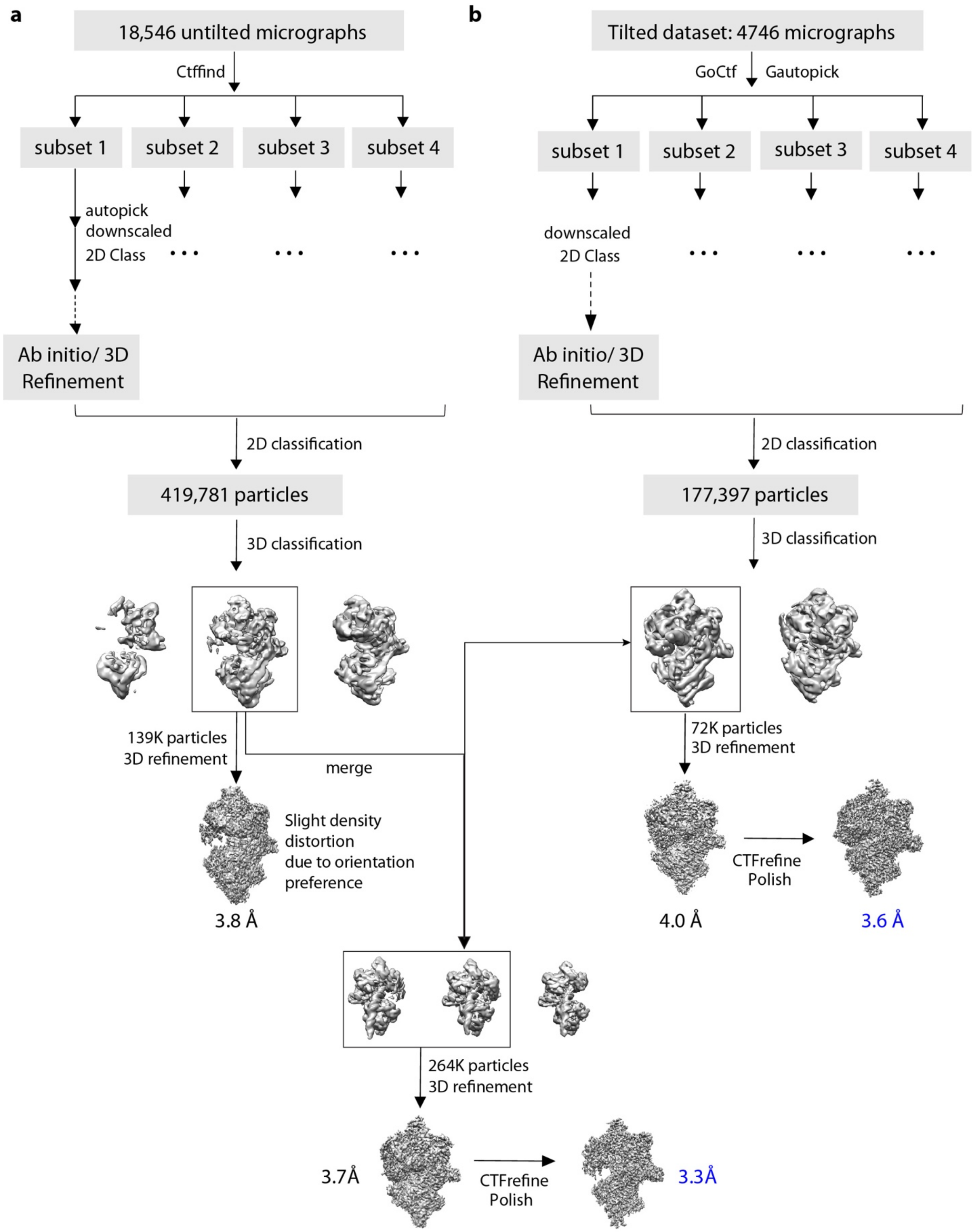

Supplementary Figure 2. Image processing procedure of the untilted and tilted datasets, leading to two final 3D maps at 3.3-Å and 3.6-Å resolution, respectively.

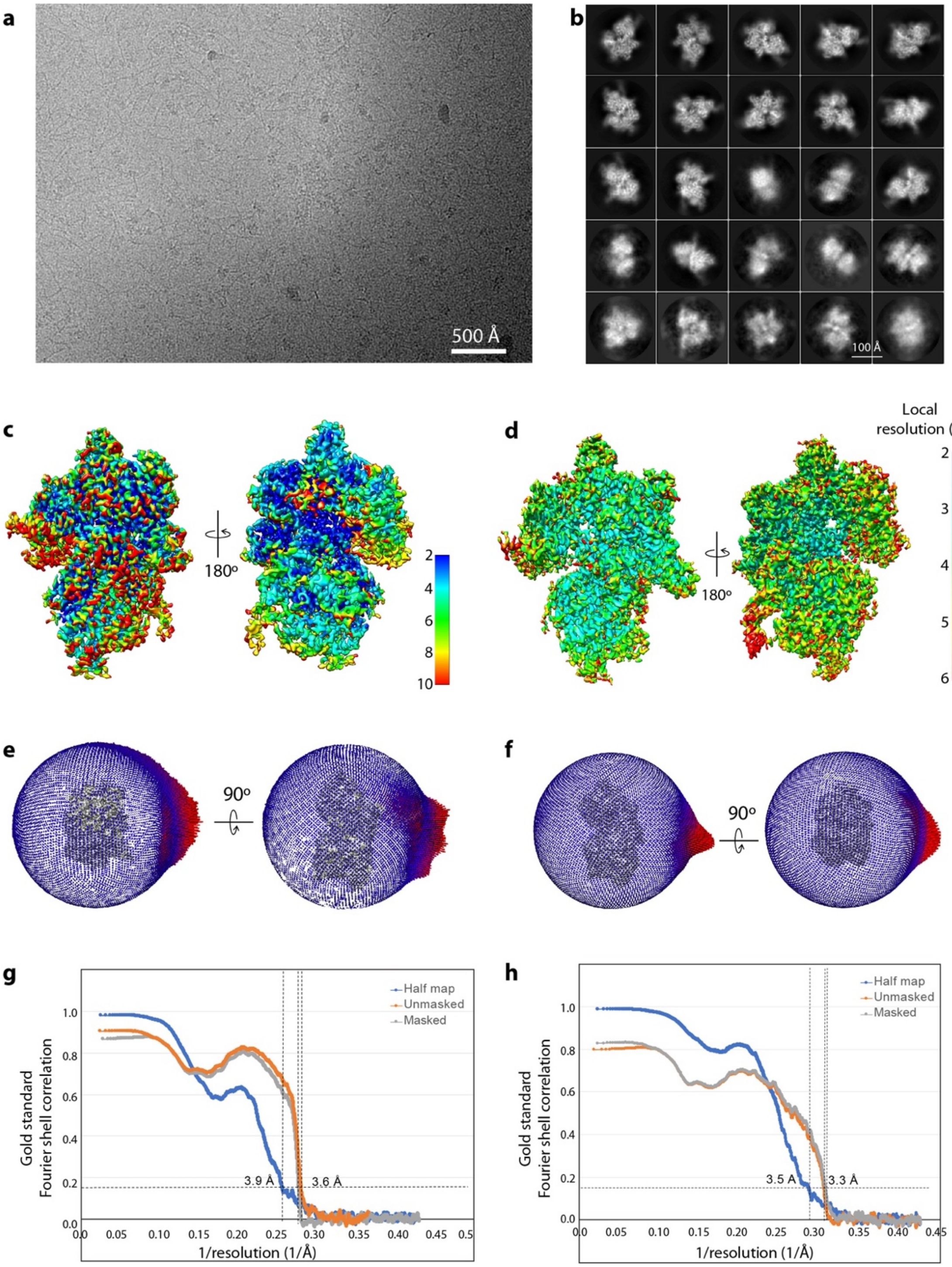

Supplementary Figure 3. Cryo-EM 3D reconstruction. a) A typical raw image. A total of 23,292 raw micrographs were recorded. **b**) The 2D class averages of the tilted dataset. **c**) The local resolution of the 3.6-Å 3D map. **d**) The local resolution of the 3.3-Å 3D map. **e**) The angular distribution of the 3.6-Å map. **f**) The angular distribution of the 3.3-Å map. **g**) The FSC curves of the 3.6-Å map. **h**) The FSC curve of the 3.3-Å map.

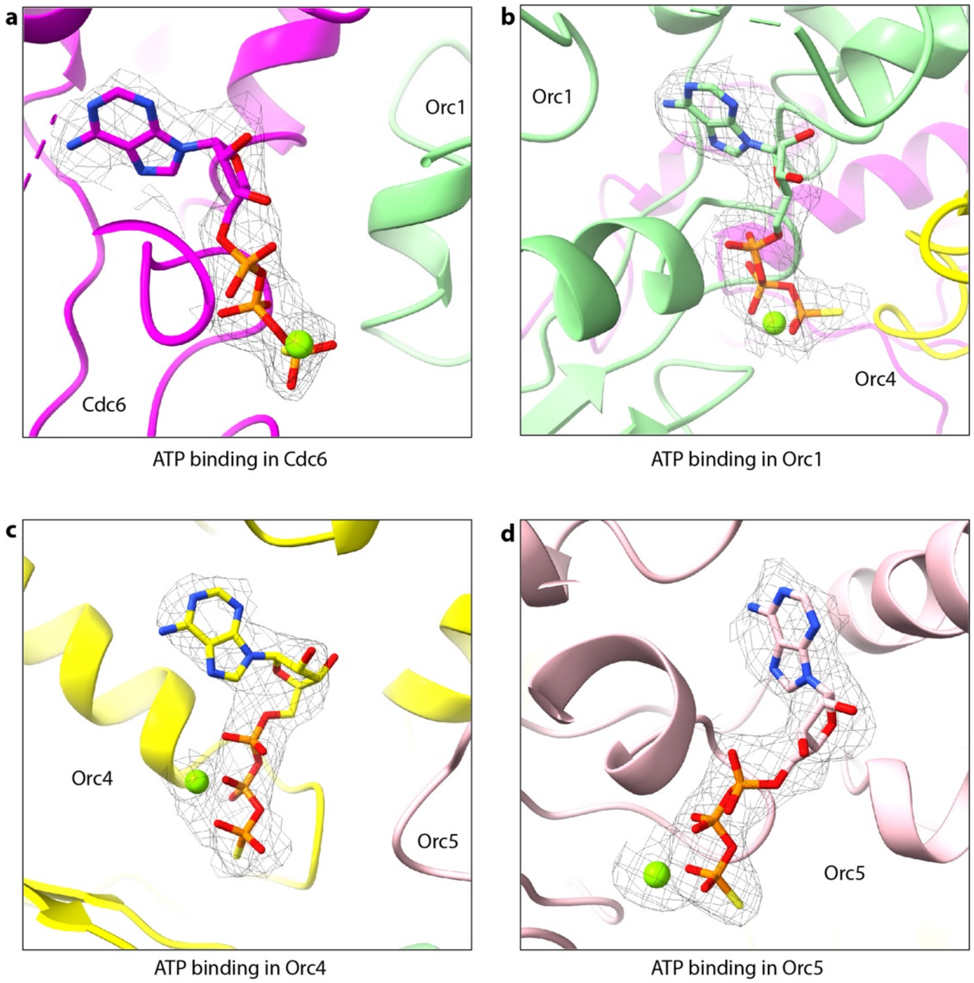

Supplementary Figure 4. The binding pockets for ATPγS in Cdc6 (a), Orc1 (b), Orc4 (c), and Orc5 (d). The bound nucleotide density is superimposed on the atomic model.

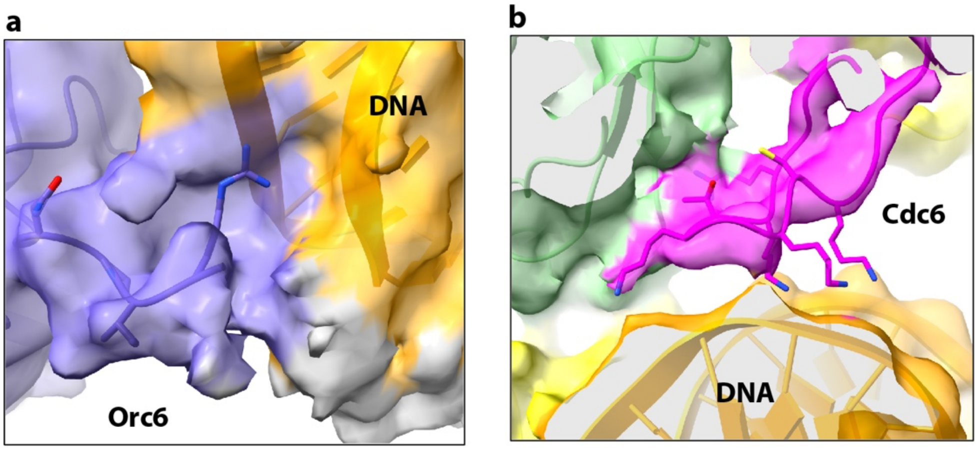

Supplementary Figure 5. Two additional DNA binding sites in the ORC–Cdc6 structure that do not exist in the ORC alone structure. a) The Orc6 basic patch interacts with DNA. **b**) The hairpin loop in the Cdc6 WHD interacts with DNA. The 3D map of ORC–Cdc6–DNA was low-pass-filtered to 4.0 Å resolution to increase the density connectivity and is rendered in surface view.

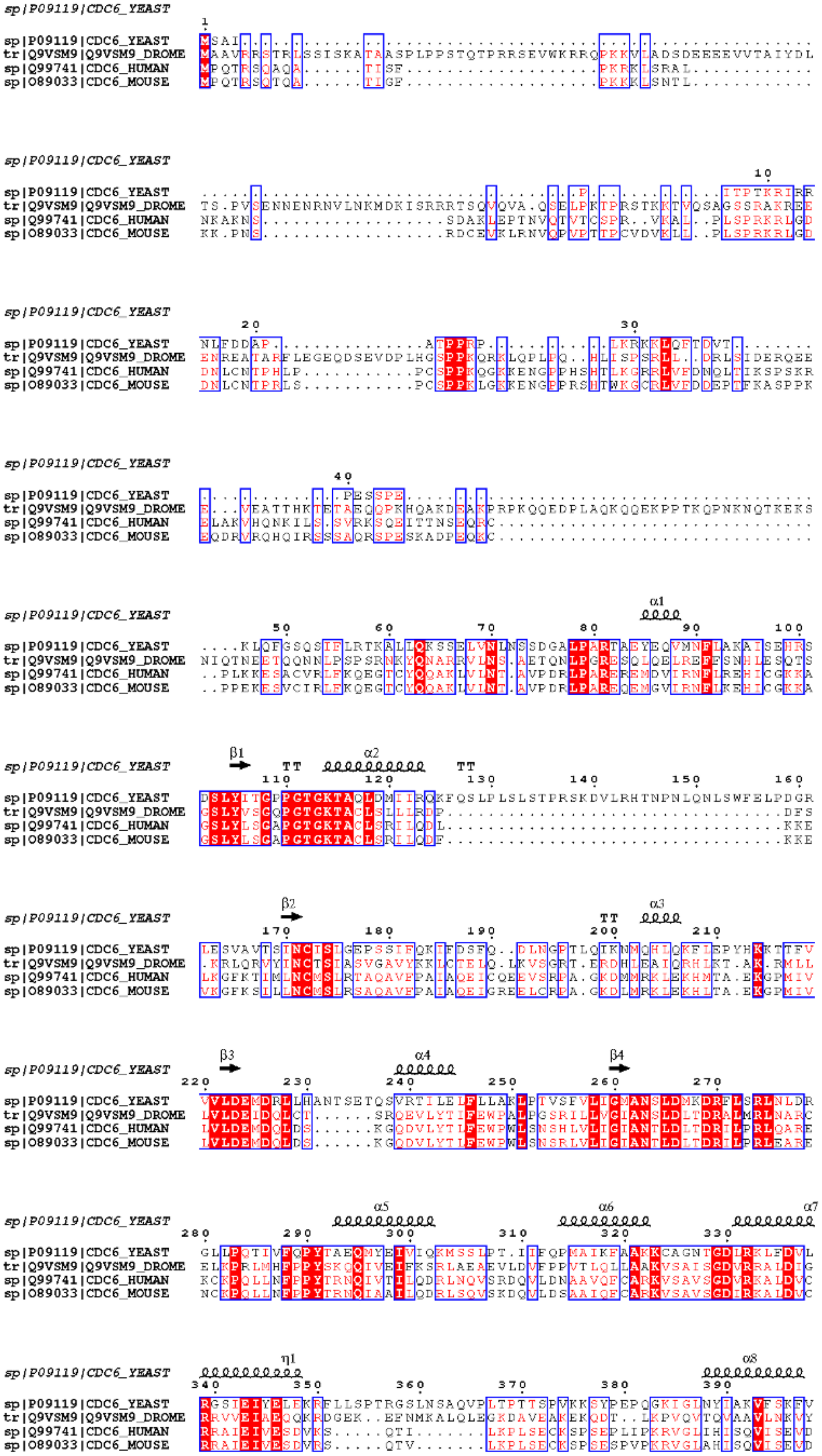
sp|P09119|CDC6_YEAST
tr|Q9VSM9|Q9VSM9_DROME
sp|Q99741|CDC6_HUMAN
sp|O89033|CDC6_MOUSE

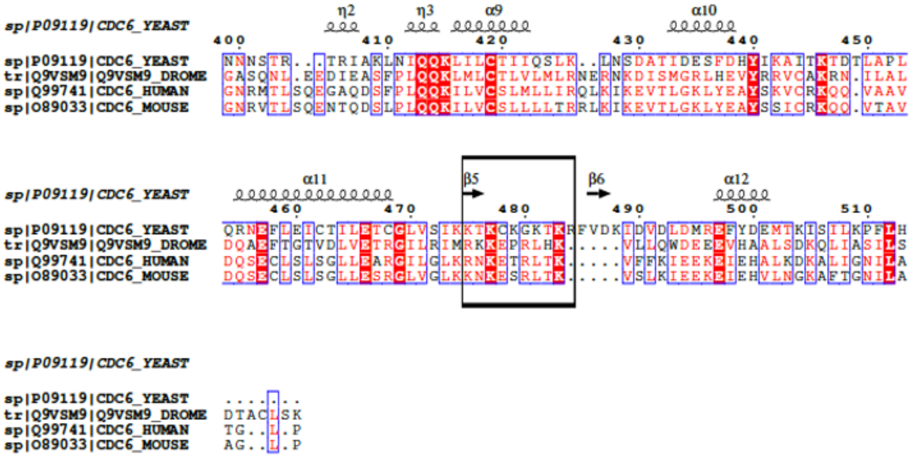

Supplementary Figure 6. Sequence alignment of Cdc6. Cdc6 sequences of *S. cerevisiae*, *Drosophila melanogaster*, human, and mouse were aligned by T-COFFEE and further analyzed by ESPript3.0. The region shown in Fig. 2d is highlighted by a black square box.

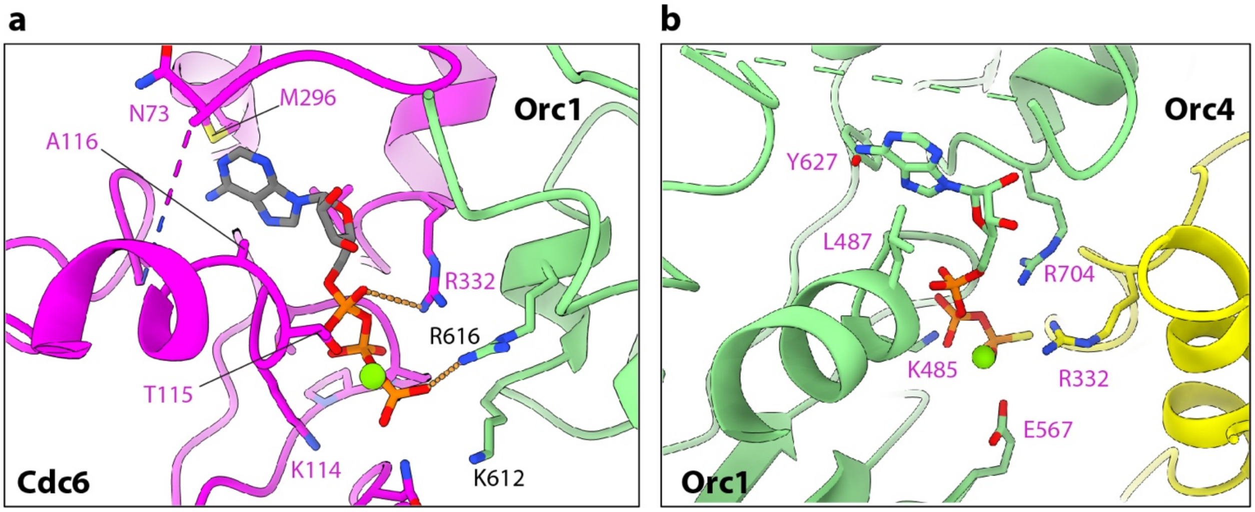

Supplementary Figure 7. Comparing the ATPγS binding pockets of Cdc6 (a) and Orc1 (b). ATPγS and the interacting residues are shown as sticks.
