# Supplementary material for: The structure of ORC–Cdc6 on an origin DNA reveals the mechanism of ORC activation by the replication initiator Cdc6
Source: Nat Commun. 2021 Jun 23;12:3883. doi: 10.1038/s41467-021-24199-1 (PMC8222357; doi:10.1038/s41467-021-24199-1)
Supplement: Supplementary file 2 — Reporting summary. [file 41467_2021_24199_MOESM2_ESM.pdf]

nature research

Corresponding author(s): Bruce Stillman, Christian Speck, Huilin Li

Last updated by author(s): May 21, 2021

Reporting Summary

Nature Research wishes to improve the reproducibility of the work that we publish. This form provides structure for consistency and transparency in reporting. For further information on Nature Research policies, see our Editorial Policies and the Editorial Policy Checklist.

Statistics

For all statistical analyses, confirm that the following items are present in the figure legend, table legend, main text, or Methods section.

n/a	Confirmed	
☐	☒	The exact sample size (n) for each experimental group/condition, given as a discrete number and unit of measurement
☐	☒	A statement on whether measurements were taken from distinct samples or whether the same sample was measured repeatedly
☒	☐	The statistical test(s) used AND whether they are one- or two-sided<br>*Only common tests should be described solely by name; describe more complex techniques in the Methods section.*
☒	☐	A description of all covariates tested
☒	☐	A description of any assumptions or corrections, such as tests of normality and adjustment for multiple comparisons
☐	☒	A full description of the statistical parameters including central tendency (e.g. means) or other basic estimates (e.g. regression coefficient) AND variation (e.g. standard deviation) or associated estimates of uncertainty (e.g. confidence intervals)
☒	☐	For null hypothesis testing, the test statistic (e.g. F, t, r) with confidence intervals, effect sizes, degrees of freedom and P value noted<br>*Give P values as exact values whenever suitable.*
☒	☐	For Bayesian analysis, information on the choice of priors and Markov chain Monte Carlo settings
☒	☐	For hierarchical and complex designs, identification of the appropriate level for tests and full reporting of outcomes
☒	☐	Estimates of effect sizes (e.g. Cohen's d, Pearson's r), indicating how they were calculated

Our web collection on statistics for biologists contains articles on many of the points above.

Software and code

Policy information about availability of computer code

Data collection	Cryo-EM data collection used SerialEM (version 3.8.8) in Titan Krios and EPU as implemented in Arctica by the manufacturer Thermo-Fisher Scientific.
Data analysis	RELIONS 3.0, MotionCorr 2.0, CTFFIND 4.1, Gautomatch (version 0.56), ChimeraX 1.0, PyEM (version 0.5), Coot (version 0.8.9.1), Phenix (version 1.14-3260), and MolProbity (version 4.5), and ImageJ (1.50i).

For manuscripts utilizing custom algorithms or software that are central to the research but not yet described in published literature, software must be made available to editors and reviewers. We strongly encourage code deposition in a community repository (e.g. GitHub). See the Nature Research guidelines for submitting code & software for further information.

Data

Policy information about availability of data

All manuscripts must include a data availability statement. This statement should provide the following information, where applicable:

- Accession codes, unique identifiers, or web links for publicly available datasets
- A list of figures that have associated raw data
- A description of any restrictions on data availability

The 3D cryo-EM maps of ORC–Cdc6–DNA at 3.3 Å and 3.6 Å have been deposited in the Electron Microscopy Data Bank under accession code EMD-23818 and EMD-23755, respectively. The atomic model based on the two 3D maps has been deposited in the Protein Data Bank under accession code PDB ID 7MCA.

Field-specific reporting

Please select the one below that is the best fit for your research. If you are not sure, read the appropriate sections before making your selection.

☒ Life sciences ☐ Behavioural & social sciences ☐ Ecological, evolutionary & environmental sciences

For a reference copy of the document with all sections, see nature.com/documents/nr-reporting-summary-flat.pdf

Life sciences study design

All studies must disclose on these points even when the disclosure is negative.

Sample size	Sample size was not redetermined. We collected two datasets (A and B). Dataset A was untilted and had 18,546 movie stacks, leading to 419,781 good particle images after 2D and 3D classification selection. Dataset B was 20 degree tilted and had 4746 movie stacks, leading to 72,000 good particle images after 2D and 3D classification selection. The data size was deemed sufficient as it resulted in 3D maps detailed enough for atomic model building.
Data exclusions	"Bad" raw particle images of the ORC-Cdc6-DNA complex that did not produce 2D class averages or 3D class maps with defined features were excluded after 2D and 3D classifications. This criteria is empirical but is a standard image processing practice in the cryoEM community.
Replication	Reproducibility resides in the large number of particles used to derive at the final 3D maps or 2D averages. The reliability and the resolution is measured by gold-standard Fourier shell correlation. Replication efforts with multiple refinement runs yielded was successful, yielding similar 3D maps.
Randomization	The raw particles were automatically selected by computer program (RELIONS 3.0).
Blinding	The investigators were not blinded to the specific data points during data collection and analysis, because visual inspection is necessary to ascertain the data quality.

Reporting for specific materials, systems and methods

We require information from authors about some types of materials, experimental systems and methods used in many studies. Here, indicate whether each material, system or method listed is relevant to your study. If you are not sure if a list item applies to your research, read the appropriate section before selecting a response.

Materials & experimental systems

n/a	Involved in the study	
☒	☐	Antibodies
☒	☐	Eukaryotic cell lines
☒	☐	Palaeontology and archaeology
☒	☐	Animals and other organisms
☒	☐	Human research participants
☒	☐	Clinical data
☒	☐	Dual use research of concern

Methods

n/a	Involved in the study	
☒	☐	ChIP-seq
☒	☐	Flow cytometry
☒	☐	MRI-based neuroimaging
